# The Serum Concentration of Vancomycin as a Diagnostic Predictor of Nephrotoxic Acute Kidney Injury in Critically Ill Patients

**DOI:** 10.3390/antibiotics11010112

**Published:** 2022-01-15

**Authors:** Welder Zamoner, Karina Zanchetta Cardoso Eid, Lais Maria Bellaver de Almeida, Isabella Gonçalves Pierri, Adriano dos Santos, André Luis Balbi, Daniela Ponce

**Affiliations:** 1 Botucatu School of Medicine, University São Paulo State—UNESP, Botucatu 18618-687, SP, Brazil; karinaeid27@gmail.com (K.Z.C.E.); bellaver.lais@gmail.com (L.M.B.d.A.); isagpierri@gmail.com (I.G.P.); andre.balbi@unesp.br (A.L.B.); daniela.ponce@unesp.br (D.P.); 2 Clinics Hospital Pharmacy, Botucatu School of Medicine, Botucatu 18618-687, SP, Brazil; adrianosantosbtu@yahoo.com.br

**Keywords:** acute kidney injury, nephrotoxicity, vancomycin, sepsis

## Abstract

The impact of serum concentrations of vancomycin is a controversial topic. Results: 182 critically ill patients were evaluated using vancomycin and 63 patients were included in the study. AKI occurred in 44.4% of patients on the sixth day of vancomycin use. Vancomycin higher than 17.53 mg/L between the second and the fourth days of use was a predictor of AKI, preceding AKI diagnosis for at least two days, with an area under the curve of 0.806 (IC 95% 0.624–0.987, *p* = 0.011). Altogether, 46.03% of patients died, and in the Cox analysis, the associated factors were age, estimated GFR, CPR, and vancomycin between the second and the fourth days. Discussion: The current 2020 guidelines recommend using Bayesian-derived AUC monitoring rather than trough concentrations. However, due to the higher number of laboratory analyses and the need for an application to calculate the AUC, many centers still use therapeutic trough levels between 15 and 20 mg/L. Conclusion: The results of this study suggest that a narrower range of serum concentration of vancomycin was a predictor of AKI in critically ill septic patients, preceding the diagnosis of AKI by at least 48 h, and it can be a useful monitoring tool when AUC cannot be used.

## 1. Introduction

The impact of the serum concentrations of vancomycin in hospitalized patients in intensive care units (ICU) is a controversial topic, and it is necessary to improve the knowledge of sepsis and the safe and effective use of vancomycin.

Sepsis is a frequent diagnosis in ICUs and corresponds to the primary etiology of acute kidney injury in this scenario with a high mortality [1,2,3,4,5]. Thus, the early administration of broad-spectrum antibiotics is justified [6].

Vancomycin is widely used in ICUs. Due to the pharmacokinetic changes of the critical patient related to the distribution, elimination, and metabolization of the drug, there is an increased risk of subtherapeutic concentrations, which may compromise the treatment and induce bacterial resistance. On the other hand, it is a drug whose main side effect is nephrotoxicity [5,6,7,8,9].

An area under the curve over the minimum inhibitory concentration (AUC/MIC) equal or greater than 400, determining the optimum activity of the antimicrobial, is usually obtained in patients with a vancomycin serum concentration in the trough of 15 to 20 mg/L. Such troughs are rarely needed to achieve this AUC target and may exceed and cause toxicity [10,11]. The association between serum vancomycin concentration and clinical outcomes is poorly studied.

## 2. Materials and Methods

The patients who entered the hospital’s ICU of Botucatu Medical School from August 2016 to July 2017 and initiated vancomycin in the preceding 48 h were included in a prospective cohort study. The quick-SOFA tool was used to identify patients with sepsis [2]. The initial dose of vancomycin was 25 mg/kg at a maintenance of 15 mg/kg. The dosing interval was 12/12 h up to 96/96 h, varying according to the serum concentration.

The following patients were excluded: patients who developed AKI in less than 48 h from the start of vancomycin use, patients with installed AKI before the introduction of vancomycin, patients with AKI of other etiologies (obstructive, vascular, glomerular, or ischemic AKI), kidney transplant, or stage V of chronic kidney disease (with estimated or measured serum creatinine clearance less than 15 mL/min), patients who were pregnant, or under 18 years old.

This study was registered in the Brazilian Registry of clinical trials (ReBEC) under Number RBR-4zrwtz and was approved by the Research Ethics Committee of Botucatu School of Medicine with number CAAE 65827117.4.0000.5411. All the research was performed following current regulations and a written informed consent statement was obtained from all participants or their legal guardians. 

The patient included in the study had his clinical and laboratory evaluation taken daily by the same observer, who consulted his electronic medical record until his treatment with vancomycin or his clinical outcome ended.

The lowest value baseline creatinine was considered in 6 months if the prior creatinine or lower creatinine were available during hospitalization since the patient was not on dialysis. KDIGO used the AKI definitions as any of the following: increase in serum creatinine by 0.3 mg/dL or more within 48 h, or increase in serum creatinine to 1.5 times baseline or more within the last 7 days, or urine output less than 0.5 mL/kg/h for 6 h [5].

The serum toxic, subtherapeutic, and therapeutic concentrations were defined as trough concentrations higher than 20 mg/L, lower than 15 mg/L, and between 15 and 20 mg/L, respectively, in two consecutive dosages.

Frequency and measures of central tendency and dispersion were calculated for the categorical and continuous variables, respectively, and established as outcome variables: AKI and death. Student’s t-test was used to compare parametric variables between two groups and the Mann–Whitney test was used for non-parametric variables. Categorical variables were compared with the chi-square or Fisher’s exact test. Variables with significant univariate associations were considered as candidates for Cox regression analysis, which was performed using backward variable selection, with the exit criteria set at *p* < 0.20.

ROC curves were built to evaluate the cutoff points of serum concentration of vancomycin as predictors of the diagnosis and prognosis of AKI in the moments until the second day (T0-2), between the second and the fourth days (T2-4), and between the fourth and the sixth days (T4-6). 

Areas under the curve (AUC) between 0.7 and 0.79 were considered a satisfactory performance and between 0.8 and 0.89 an optimum performance.

Using Lee Formula for an alpha error of 0.05 and study power of 80%, the sample size calculation was performed, and considering that the prevalence of the AKI outcome would be 30% higher in patients with a toxic concentration of vancomycin [12], 30 patients were estimated with toxic concentrations and 30 without toxic concentrations (total of 60 patients).

## 3. Results

**Figure 1:**
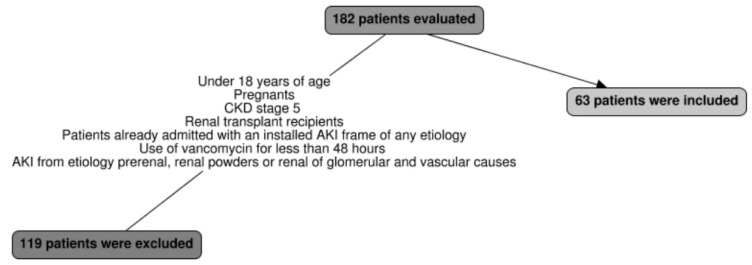
Flow diagram of patients included and excluded.

We evaluated 63 patients (Figure 1), aged 54.67 ± 18.7 years, with a male predominance (66.7%), BMI 26.1 ± 6.8, and the use of vancomycin for 11.4 ± 7.33 days. The vast majority of patients (92%) had performed the serum vancomycin measurement, the number of serum concentrations dosages and the number of posologic adjustments were 3.87 and 1.84, respectively; 53.96% were in concentrations considered toxic (higher than 20 mg/L), an average of 25.5 ± 11.90 mg/L. AKI prevalence was 44.4%, with the stage KDIGO 3 being most common (46.4%), and 46% died.

Clinical and laboratory variables are shown in Table 1, and vancomycin characteristics are shown in Table 2, distinguishing patients who developed AKI or not.

The only variable identified as a risk factor for AKI was the vancomycin concentration between the second and the fourth days (T2-4) in the Cox regression analysis (HR = 1.086, *p* = 0.009), without statistically significant differences in other variables, as shown in Table 3.

**Figure 2:**
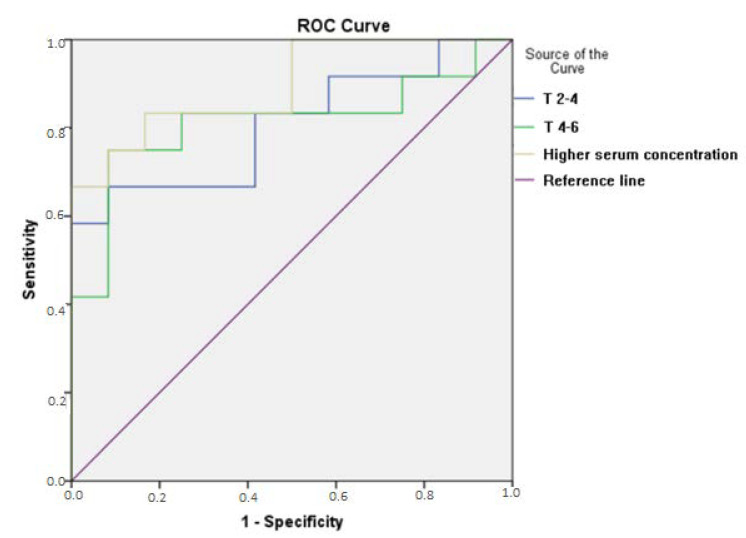
ROC curve for the AKI outcome in the population of septic patients using vancomycin admitted to the ICU. T2-4: Serum concentration between the second and fourth day of use of vancomycin (48–96 h); T4-6: Serum concentration between the fourth and sixth day of use of vancomycin (96–144 h).

Vancomycin serum concentration in T2-4 days higher than 17.53 was a predictor of AKI with a sensitivity of 79.7% and specificity of 83.3% by ROC curve analysis, with an AUC of 0.806 (IC 95% 0.624–0.987, *p* = 0.011), as shown in Figure 2. AKI occurred on average on the sixth day of vancomycin use, and the founded value preceded the diagnosis of AKI by two days.

**Figure 3:**
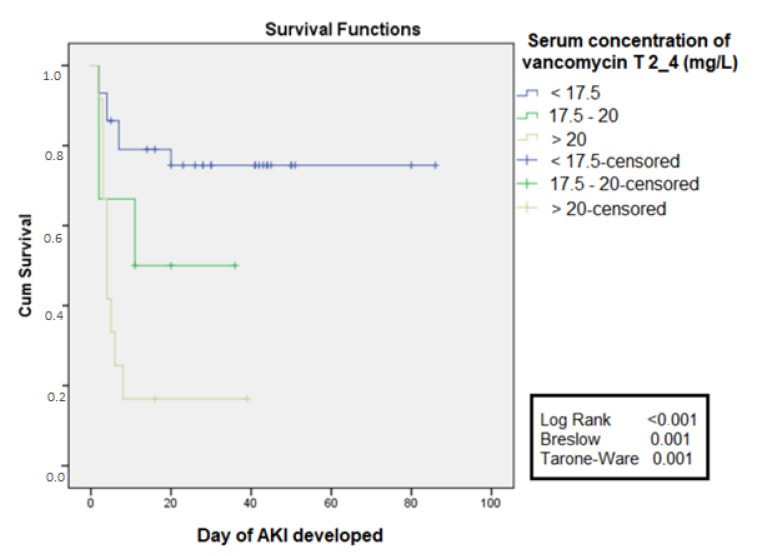
Free time for acute kidney injury according to the values of serum concentration between T2-4 days (48–96 h) use of vancomycin in septic patients in ICUs.

Based on the values of cutoff obtained by the ROC curve at 2 to 4 days, the free time curve for AKI was constructed. It was observed that in the group with serum concentration >20 mg/L the free time for the development of AKI was lower when compared to the group that had serum concentrations between 17.5 and 20 mg/L, which also presented shorter free time compared to the group with a serum concentration <17.5 mg/L, log-rank <0.001 (Figure 3).

Table 4 shows the clinical and laboratory variables, and Table 5 shows the characteristics related to vancomycin, distinguishing patients who died or survived.

When the two groups were analyzed using Cox regression, it was observed that the variables age (HR = 1.13, *p*= 0.018), glomerular filtration rate estimated by CKD-EPI (HR 1.23, *p* = 0.015), levels of serum concentration at the moment 2 to 4 days (HR = 1.60, *p* = 0.021) and mean C-protein reactive value (HR 1.26, *p* = 0.011) were identified as risk factors for death, as shown in Table 6.

**Figure 4:**
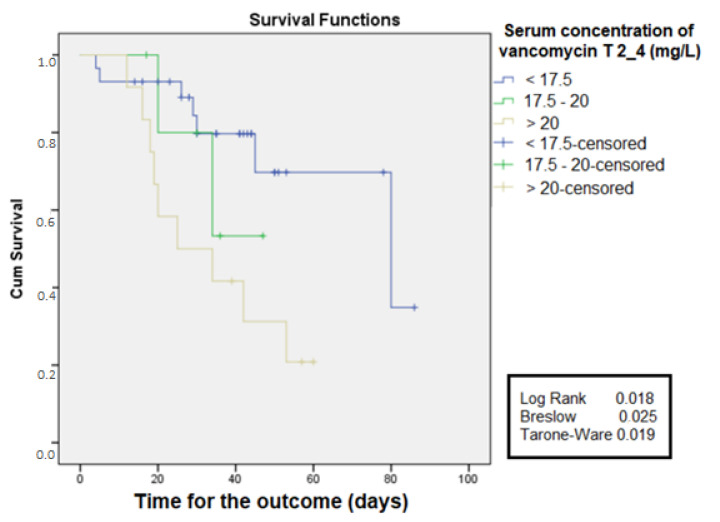
Free time for the outcome of death according to values of serum concentration between T2-4 days (48–96 h) of vancomycin use in ICU patients.

Based on the values of cutoff obtained by the ROC curve at 2 to 4 days, the free time curve for death was constructed. It was observed that, in the group with serum concentration >20 mg/L, the free time for the development of AKI was lower when compared to the group that had serum concentrations between 17.5 and 20 mg/L, which also presented a lower free time compared to the group with a serum level <17.5 mg/L and log-rank 0.018, as shown in Figure 4.

## 4. Discussion

Due to the pharmacokinetic changes of the critical patient related to the drug distribution, elimination, and metabolization, two concerns still permeate vancomycin use, related to its efficacy and safety. There is an increased risk of subtherapeutic concentrations, which may compromise the treatment and induce bacterial resistance. On the other hand, it is a drug whose main side effect is nephrotoxicity, with AKI risk and short- and long-term problems. 

This study assessed the impact of therapeutic monitoring of vancomycin on clinical outcomes. It is known that the serum concentration of vancomycin in the trough between 15 and 20 mg/L corresponds to an area under the curve over the minimum inhibitory concentration (AUC/MIC) equal or greater than 400, determining the optimum activity of the antimicrobial [10,11]. However, the association between serum vancomycin concentration and clinical outcomes is poorly studied.

Were evaluated 182 critically ill patients using vancomycin, and 63 patients were included in the study. This difficulty of studying vancomycin nephrotoxicity arises from the following problem: high serum levels are a consequence or cause of AKI due to the accumulation of the drug, which is caused by a reduction in its renal whitening due to septic AKI, as approached by Álvarez et al. [12] and by American guideline [11]. Therefore, the presence of AKI already installed or started before 48 h of vancomycin use was an exclusion criteria for this study.

Most critically ill patients (92%) had performed the serum vancomycin measurement, and the mean measurements of serum concentrations and dose adjustments were 3.87 and 1.84, respectively. Considering that the average time of use was 11.43 days, and according to the protocol already established in the literature, there is an indication that it is not always serum concentrations determined and adjusted by the ICU team, which can contribute to subtherapeutic concentrations of the antimicrobial. Iwamoto et al. [13] found an increased risk of AKI and nephrotoxicity in patients who were not submitted to the monitoring of serum concentrations of vancomycin (OR = 0.25 and *p* < 0.05).

A study conducted by Davis et al. [14] assessed adherence to the guidelines established by the American consensus [11] and showed that only 19% of the institutions questioned used a standard definition to identify nephrotoxicity associated with vancomycin. The most current 2020 guidelines recommend using Bayesian-derived AUC monitoring rather than trough concentrations [14]. 

Despite the availability of therapeutic drug monitoring, there is a difficulty in achieving and maintaining adequate serum concentrations, especially in the intensive care environment, due to the collection, the patient, and the pharmacokinetic variation of the drugs [8]. In our study, this difficulty also occurred, with high rates of toxic and subtherapeutic concentration. 

AKI occurred in 44.4% of patients, with a mortality rate of 46%. On average, the development of AKI occurred on the sixth day of vancomycin use, compatible with data from the literature in which nephrotoxic AKI generally occurs from 4 to 8 days after the initiation of treatment [15,16]. Such data indicate that it was possible to evaluate the role of the nephrotoxicity of vancomycin as the cause of AKI in septic patients in ICUs. Since the role of sepsis was more critical than that of nephrotoxicity, the incidence of AKI (close to 60%), as well as mortality (higher than 70%), would be higher.

The only variable that showed an association with AKI by Cox regression analysis was the highest level of serum vancomycin between the second and fourth days. It differs from previous studies that identified other variables (use of vasoactive drugs, basal creatinine, and age) as predictors of AKI in ICU patients [17,18]. In our study, we evaluated a specific population using vancomycin after ICU admission, and patients with AKI were excluded before introducing the antimicrobial agent, which could justify the different risk factors identified in other studies. 

Serum concentrations above 17.53 mg/L between the second and fourth day of use were an excellent predictor of AKI in the critical population, with AUC higher than 0.8 and sensitivity and specificity close to 80%, preceding the diagnosis of AKI in at least 48 h. However, this value is within the range considered therapeutic for severe infections (15–20 mg/L), suggesting that therapeutic concentrations should be less in the critical population due to the presence of other risk factors for AKI: advanced age, previously diminished renal function, dehydration, and the duration of sepsis; the concomitant administration with other nephrotoxic drugs, such as amphotericin B, aminoglycosides, intravenous contrast way, and loop diuretics; and the need for vasopressors due to hemodynamic instability [19,20]. A free time curve was built for AKI, stratifying the serum concentration levels in less than 17.5 mg/L, between 17.5 mg/L and 20 mg/L, and higher than 20 mg/L, showing the shorter free time and higher serum levels, with a significant difference between curves. 

Bosso et al. [15] evaluated 288 patients in a prospective multicentric study and found AKI in 29.6% of patients with serum concentrations levels higher than 15 mg/L and in 8.9% of the patients with vancomycin concentrations lower than 15 mg/L. Gupta et al. [19] identified AKI incidence in 27% of patients, with vancomycin concentrations higher than 15 mg/L as a predictor of nephrotoxicity. Thus, it is questionable whether the ideal therapeutic concentrations aim to prevent AKI.

Age, mean PCR value, the serum concentration of vancomycin between the second and fourth days, and estimated glomerular filtration rate were associated with mortality. Chertow et al. [20] showed that small increases in serum creatinine were significantly associated with the increase in AKI patients’ mortality. Liangos et al. [21] found that chronic diseases such as diabetes melittus (DM) were associated with a higher risk of development of AKI, with a consequent increase in mortality. CPR concentration indicating systemic inflammation was shown in the literature associated with mortality, as presented by Villacorta et al. [22] in patients with heart failure, in which CPR > 3 mg/dL was associated with a higher mortality in comparison to individuals with lower values (*p* = 0.018). 

This study presents some limitations: the obtained sample was small due to the difficulty of studying nephrotoxicity in critically ill patients, since there are many exclusion variables; the data were obtained in a single center; the agents’ resistance to vancomycin was not studied; and the serum concentration levels were not studied as a prognostic predictor of AKI (severity and need for acute renal support). Despite these limitations, this was the first study of the therapeutic monitoring of vancomycin to present cutoff values to refine the management in the population of septic patients in an intensive care scenario, when AUC cannot be used.

## 5. Conclusions

The current 2020 guidelines [23] recommend using Bayesian-derived AUC monitoring rather than trough concentrations. However, due to a higher number of laboratory analyses and the need for an application to calculate the AUC, many centers still use therapeutic trough levels between 15 and 20 mg/L. The results of this study suggest that a narrower range of serum concentration of vancomycin was a predictor of AKI in critically ill septic patients, preceding the diagnosis of AKI in at least 48 h, and can be a useful monitoring tool when AUC cannot be used.

When the early identification of serum vancomycin levels is performed, it is possible to make dose adjustments essential to prevent AKI or modify its natural history.

## Figures and Tables

**Table 1:** Clinical and laboratory characteristics of patients hospitalized in an intensive care unit, using vancomycin for the presence or absence of AKI.

Variable	General	With AKI	Without AKI	*p*-Value
	(N = 63)	(N = 28)	(N = 35)	
Age (years) *	54.6 ± 18.7	58.1 ± 17.2	51.9 ± 19.7	0.19
Male	42 (66.7)	19 (67.8)	23 (65.7)	0.89
Weight (kg) *	72.6 ± 18.6	71.4 ± 17.05	73.5 ± 19.9	0.66
GFR baseline—CKD-EPI (mL/min) *	108.9 ± 28.9	98.4 ± 29.3	117.3 ± 26.1	0.009
Urinary Output (mL/kg/h) *	1.1 ± 0.5	1.08 ± 0.5	1.1 ± 0.5	0.50
CPR (mg/dL) **	16.4 (11.2–25.9)	16.2 (11.7–25.8)	12.94 (6.4–23.6)	0.13
Mean arterial pressure *	105.9 ± 11.9	78.7 ± 8.7	80.8 ± 9.3	0.37
Albumin (g/dL) *	2.25 ± 0.38	2.18 ± 0.3	2.32 ± 0.44	0.027
SOFA	8.5 ± 5.95	9.5 ± 5.54	7.0 ± 7.07	0.81
Hospitalization days *	36.1 ± 21.1	33.5 ± 20.5	30 ± 20.2	0.71
Arterial hypertension (%)	32 (50.8)	14 (40)	18 (51.4)	0.88
Diabetes Mellitus (%)	18 (28.6)	8 (28.5)	10 (28.5)	0.77
Chronic kidney disease (%)	3 (4.8)	2 (7.1)	1 (2.8)	0.58
Cardiovascular disease (%)	7 (11.1)	4 (14.2)	3 (8.5)	0.69
Mechanical Ventilation (%)	51 (81)	25 (89.2)	26 (74.2)	0.24
Vasoactive drug (%)	35 (55.6)	20 (71.4)	15 (42.8)	0.04
Contrast (%)	7 (11.1)	3 (10.7)	4 (11.4)	1.00
Diuretic (%)	24 (38.1)	15 (53.5)	9 (25.7)	0.04
Other nephrotoxic drugs (%) ^#^	5 (7.9)	3 (10.7)	2 (5.7)	0.65
Diagnosis of hospitalization (%)				
Infection	22 (34.9)	12 (42.8)	10 (28.5)	0.36
Cardiovascular	4 (6.3)	3 (10.7)	1 (2.8)	0.31
Postoperative	22 (34.9)	7 (25)	15 (42.8)	0.22
Other	15 (23.8)	6 (21.43)	9 (25.7)	0.92
Death (%)	29 (46)	15 (53.5)	14 (40)	0.34

KDIGO: Clinical Practice Guideline for Acute Kidney Injury; AKI: Acute kidney injury; CPR: C-protein reactive; GFR: glomerular filtration rate estimated by CKD-EPI; * Average and standard deviation; ** Median and quartiles; # Non-hormonal anti-inflammatory, amphotericin B, Acyclovir, amikacin.

**Table 2:** Features related to vancomycin in patients hospitalized in an intensive care unit for the presence or absence of AKI.

Variables	General	With AKI	Without AKI	*p*-Value
	(N = 63)	(N = 28)	(N = 35)	
Vancomycin use time (days) **	11.4 (8.1–15.2)	10 (7.5–15)	9 (6–13.7)	0.25
Attack dose (mg/kg) *	24.8 ± 5	24.42 ± 5.15	25.13 ± 4.93	0.58
Vancomycin doses (First) (mg/kg) **	15.3 (12.9–19.4)	15.2 (13.3–18.3)	15.7 (14.6–19.2)	0.21
Number of doses adjustments **	1.8 (0–3)	1 (0.5–3)	2 (0–3)	0.88
Toxic serum level (%)	34 (54)	22 (78.5)	15 (42.8)	0.009
Subtherapeutic level (%)	32 (50.7)	12 (42.8)	30 (85.7)	<0.001
Vancomycin T0-T2 (mg/L) *	16.08 ± 8.3	17.4 ± 11.7	14.7 ± 5.5	0.73
Vancomycin T2-T4 (mg/L) **	19.1 (7.1–26.6)	22.1 (9.1–29.6)	9.1 (6.03–13.4)	0.002
Vancomycin T4-T6 (mg/L) *	17.1 ± 8.9	20.4 ± 11.04	14.02 ± 5.08	0.051

T0-T2: Serum concentration between the beginning and the second day of use of vancomycin (0–48 h); T2-T4: serum concentration between the second and fourth day of use of vancomycin (48–96 h); T4-T6: serum concentration between the fourth and sixth day of use of vancomycin (96–144 h); AKI: Acute Kidney Injury; * Average and standard deviation; ** Median and quartiles.

**Table 3:** Cox regression of the associated variables with AKI in patients using vancomycin admitted to the ICU.

Variables	HR	Confidence Interval	*p*-Value
Albumin (g/dL)	0.49	0.12–1.91	0.3
Estimated GFR (mL/min)	0.99	0.97–1.017	0.74
Vasoactive drug	0.59	0.16–2.2	0.43
Diuretic use	1.004	0.27–3.71	0.99
Serum concentration T2-T4 (mg/L)	1.086	1.02–1.15	0.009

AKI: acute kidney injury; ICU: intensive care unit; GFR: glomerular filtration rate (CKD-EPI); T2-T4: Serum concentration between the second and fourth day of use of vancomycin (48–96 h).

**Table 4:** Patients’ clinical and laboratory characteristics in the use of vancomycin hospitalized in an intensive care unit, compared to its outcome.

Variables	General	Death	No Death	*p*-Value
	(N = 63)	(N = 29)	(N = 34)	
Age (years) *	54.6 ± 18.7	64.5 ± 17.04	46.2 ± 15.9	0.0001
Male sex (%)	42 (66.7)	19 (65.5)	23 (67.6)	1
Weight (kg) *	72.6 ± 18.6	69.1 ± 15.5	75.5 ± 20.6	0.17
GFR baseline—CKD-EPI (mL/min) *	108.9 ± 28.9	96.08 ± 31.7	119.9 ± 21.3	0.001
Diuresis mL/kg/h *	1.1 ± 0.5	1.04 ± 0.5	1.2 ± 0.5	0.17
CPR (mg/dL) *	16.4 ± 9.8	20.6 ± 10.8	12.8 ± 7.3	0.002
Mean arterial pressure (mmHg) *	105.9 ± 11.9	103.6 ± 12.3	107.8 ± 11.4	0.16
Albumin (g/dL) *	2.25 ± 0.38	2.19 ± 0.32	2.31 ± 0.43	0.19
SOFA	8.5 ± 5.95	8.88 ± 6.68	7.0 ± 0.00	0.008
Hospitalization days *	36.1 ± 21.1	30.28 ± 23.6	41.09 ± 17.6	0.04
Arterial hypertension (%)	32 (50.8)	17 (58.6)	15 (44.1)	0.37
Diabetes mellitus (%)	18 (28.6)	9 (31)	9 (26.5)	0.90
Chronic kidney disease (%)	3 (4.8)	3 (10.3)	0 (0)	0.18
Cardiovascular disease (%)	7 (11.1)	4 (13.8)	3 (8.8)	0.82
Mechanical ventilation (%)	51 (81)	24 (82.8)	27 (79.4)	0.98
Vasoactive drug (%)	35 (55.6)	17 (58.6)	18 (52.9)	0.84
Contrast (%)	7 (11.1)	4 (13.8)	3 (8.8)	0.82
Diuretic use (%)	24 (38.1)	16 (55.2)	8 (23.5)	0.02
Other nephrotoxic drugs (%)^#^	5 (7.9)	1 (3.4)	4 (11.8)	0.45
Hospitalization Diagnosis:				
Infection (%)	22 (34.9)	13 (44.8)	9 (26.4)	0.21
Cardiovascular (%)	4 (6.3)	3 (10.3)	1 (2.9)	0.32
Infection Focus (%)				
Urinary + bloodstream	4 (13.79)	4 (13.79)	0 (0)	0.04
Pulmonary	32 (50.8)	12 (41.38)	21 (61.76)	0.17
AKI (%)	28 (44.4)	15 (51.7)	13 (38.12)	0.34
KDIGO (%)				
1	5 (17.8)	3 (20)	2 (15)	0.99
2	10 (35.7)	6 (40)	4 (30)	0.76
3	13 (46.4)	6 (40)	7 (53)	0.72

KDIGO: Clinical Practice Guideline for Acute Kidney Injury; AKI: Acute kidney injury; CPR: C- protein reactive; TFG: glomerular filtration rate estimated by CKD-EPI; * Average and standard deviation; # anti-inflammatory non-hormonal, amphotericin B, Acyclovir, amikacin.

**Table 5:** Features related to vancomycin in patients hospitalized in an intensive care unit, compared to its outcome.

Variables	General	Death	No Death	*p*-Value
	(N = 63)	(N = 29)	(N = 34)	
Vancomycin use time (days) *	11.4 ± 7.3	9.4 ± 5.9	13.1 ± 8.05	0.04
Attack dose mg/kg *	24.8 ± 5	24.9 ± 4.4	24.7 ± 5.5	0.33
Vancomycin dose (First) mg/kg *	15.3 ± 3.5	14.8 ± 2.6	18.1 ± 4.1	0.90
Number of doses adjustments **	1.84 (1.8)	1.07 (1.2)	2.50 (2.07)	0.002
Toxic serum level (%)	34 (54)	16 (55.2)	18 (52.9)	1.00
Subtherapeutic level (%)	39 (61.9)	14 (48.3)	25 (73.5)	0.07
Vancomycin T0-T2 (mg/L) *	16.08 ± 8.3	17.91 ± 11.02	14.2 ± 6.5	0.65
Vancomycin T2-T4 (mg/L) *	17.6 ± 9.5	20.0 ± 9.76	11.3 ± 7.81	0.02
Vancocycin T4-T6 (mg/L) *	17.1 ±8.9	20 ± 9.07	14.08 ± 8.05	0.07

T0-T2: Serum concentration between the beginning and the second day of use of vancomycin (0–48 h); T2-T4: Serum concentration between the second and fourth day of use of vancomycin (48–96 h); T4-T6: Serum concentration between the fourth and sixth day of use of vancomycin (96–144 h); * Average and standard deviation; ** Median and quartiles.

**Table 6:** Cox regression of the associated variables to death in patients using vancomycin admitted to the ICU.

Variables	HR	Confidence Interval	*p*-Value
Age (years)	1.13	1.02–1.26	0.018
Estimated GFR (mL/min)	1.23	1.04–1.45	0.015
Number of doses adjustments	3.21	0.94–10.9	0.06
Vasoactive drug	30.68	0.37–2502.8	0.127
Vancomycin T2-T4 (mg/L)	1.60	1.07–2.4	0.021
Diuretic use	0.153	0.005–4.25	0.26
CPR (mg/dL)	1.26	1.05–1.51	0.011

ICU: intensive care unit; CPR: C-Protein Reactive; GFR: glomerular filtration rate estimated by CKD-EPI; T2-T4: Serum concentration between the second and fourth day of use of vancomycin (48–96 h).

## Data Availability

All Research Data is found in this manuscript.
